# Supplementary material for: Structural features discriminating hybrid histidine kinase Rec domains from response regulator homologs
Source: Nat Commun. 2023 Mar 2;14:1002. doi: 10.1038/s41467-023-36597-8 (PMC9981736; doi:10.1038/s41467-023-36597-8)
Supplement: Supplementary file 2 — Supplementary Information [file 41467_2023_36597_MOESM2_ESM.pdf]

Supplementary Information
for

Structural features discriminating hybrid histidine kinase Rec domains from response regulator homologs

Mitchell Brüderlin[1], Raphael Böhm[1], Firas Fadel[1], Sebastian Hiller[1], Tilman Schirmer[1*] and Badri N. Dubey[1,2*]

[1] Structural Biology, Biozentrum, University of Basel, Spitalstr. 41, 4056 Basel, Switzerland

[2] CSSB Centre for Structural Systems Biology, Deutsches Elektronen-Synchrotron DESY, Notkestr. 85, 22607 Hamburg, Germany

Correspondence: tilman.schirmer@unibas.ch, badri.nath.dubey@desy.de

Supplementary Figures

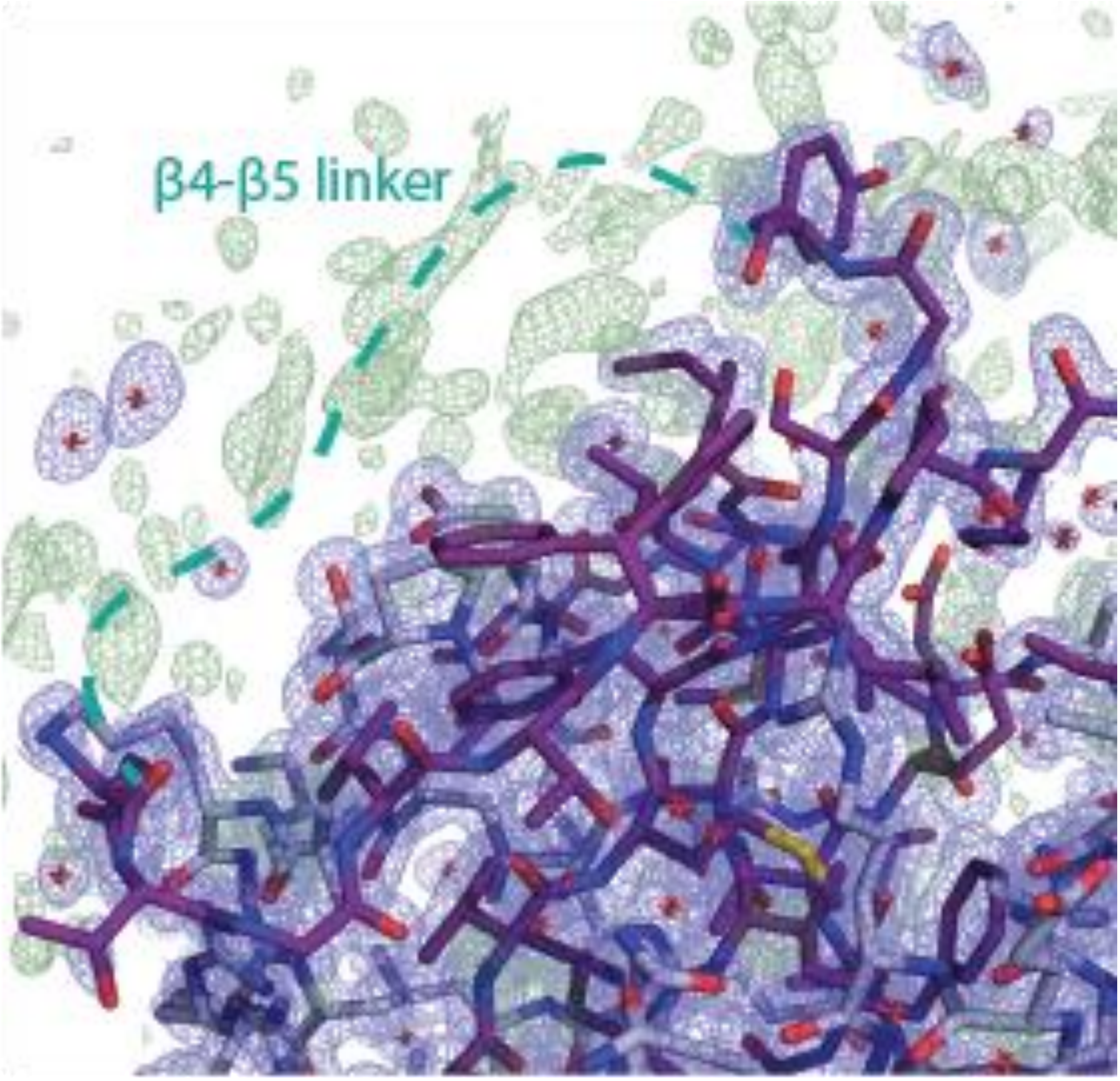

Supplementary Fig. 1. Part of CckA^{Rec} electron density and model· $2F_o$-F_c map in blue contoured at 1σ and F_o-F_c map in green contoured at 3 σ are superimposed on the CckA^{Rec} model (stick representation with carbon, oxygen and nitrogen atoms in puple, blue, and red respectevely. The electron density is clearly defined for the entire molecule with the exception of the β4 - β5 linker (dotted line) which only displays only weak and discontinues difference density (green blobs). Water molecules are shown as red crosses.

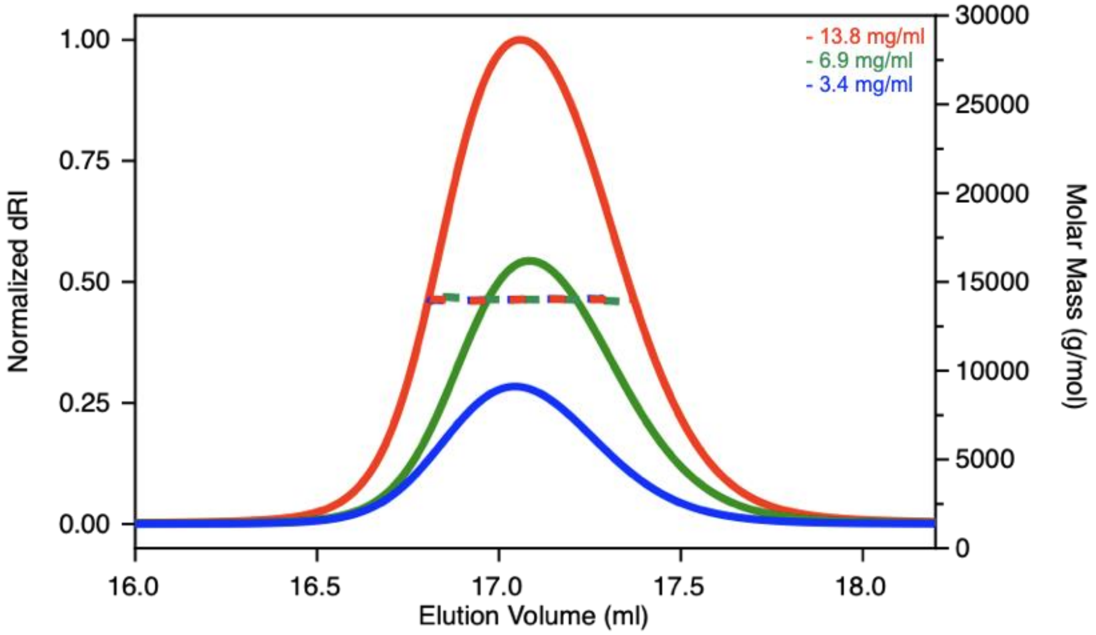

Supplementary Fig. 2. SEC-MALS profile of CckA^{Rec}. Molecular mass values (right axis) are represented by dotted lines and change in refractive index (left axis) by solid lines. Protein concentrations are shown at the top right of the figure.

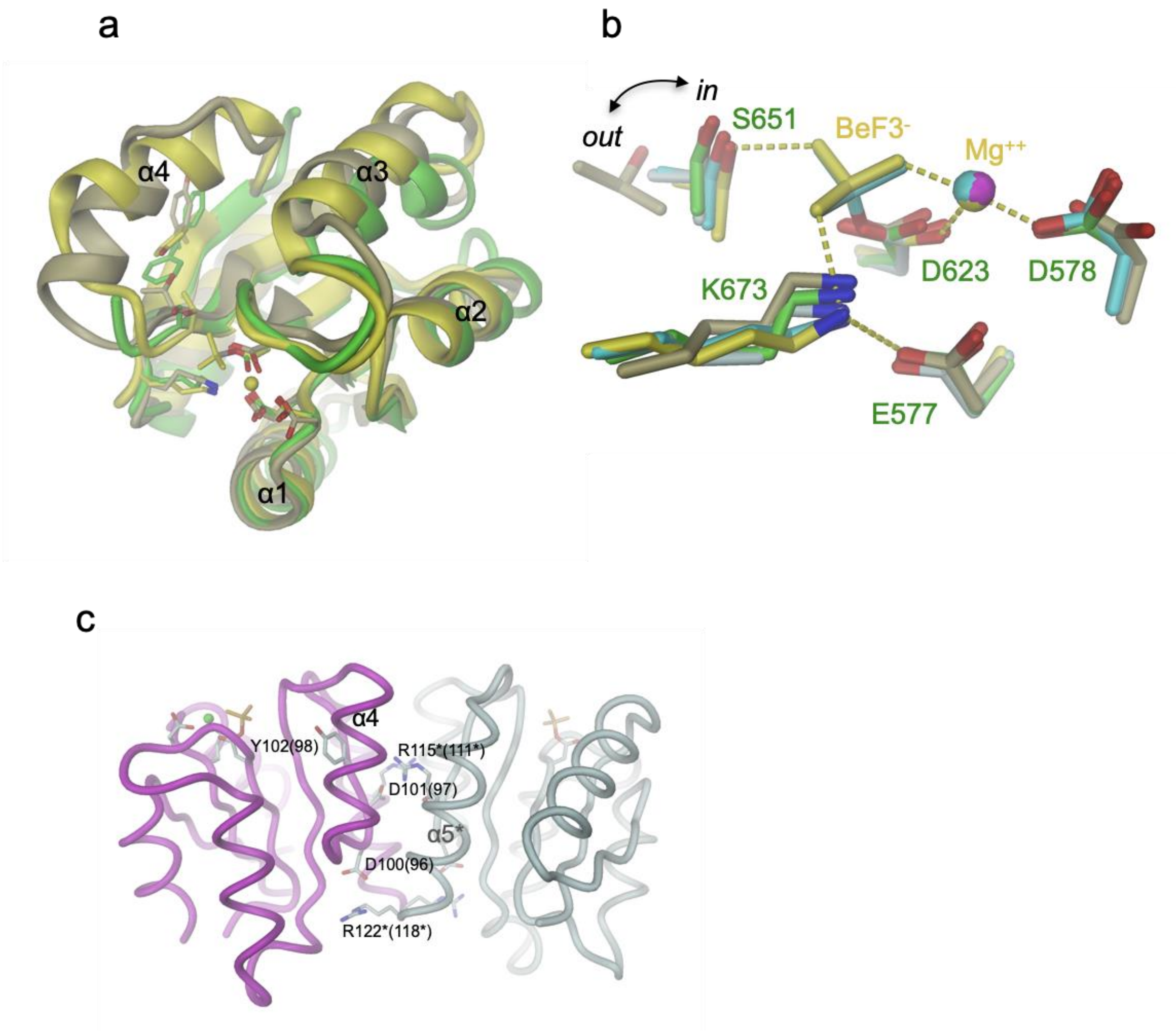

Supplementary Fig. 3. Structure comparison of CckA^{Rec} with native and activated Rec domains.

(a) Superposition of CckA^{Rec} (green), native PhoB (1b00, khaki), and BeF_3^- modified PhoB (1zes, yellow). Residues of the active site and the Y/F residue of the switch are shown in full. Note that F670 of CckA^{Rec} exhibits two alternative conformations that coincide with the outward and inward orientation seen in native and activated PhoB, respectively. For the superposition, only the Cα - positions of the depicted residues except Y/F were used.

(b) Close-up of active sites shown in (A) together with those of native (6is2, white) and BeF_3^- modified ArlR (6is1, cyan). Note that all structures, including that of CckA^{Rec} (green) exhibit the same arrangement (activated conformation, rmsd = 0.40 - 0.58 Å for all atoms) with the exception of native PhoB (khaki), which has the S/T residue in an "out" position (rmsd = 0.77 Å).

(c) Dimeric structure of BeF_3^- modified PhoB (1zes) with selected residues, including conserved charged residues of the interface, in full. Asterisks indicate symmetry related residues. CtrA residue numbers are given in brackets.

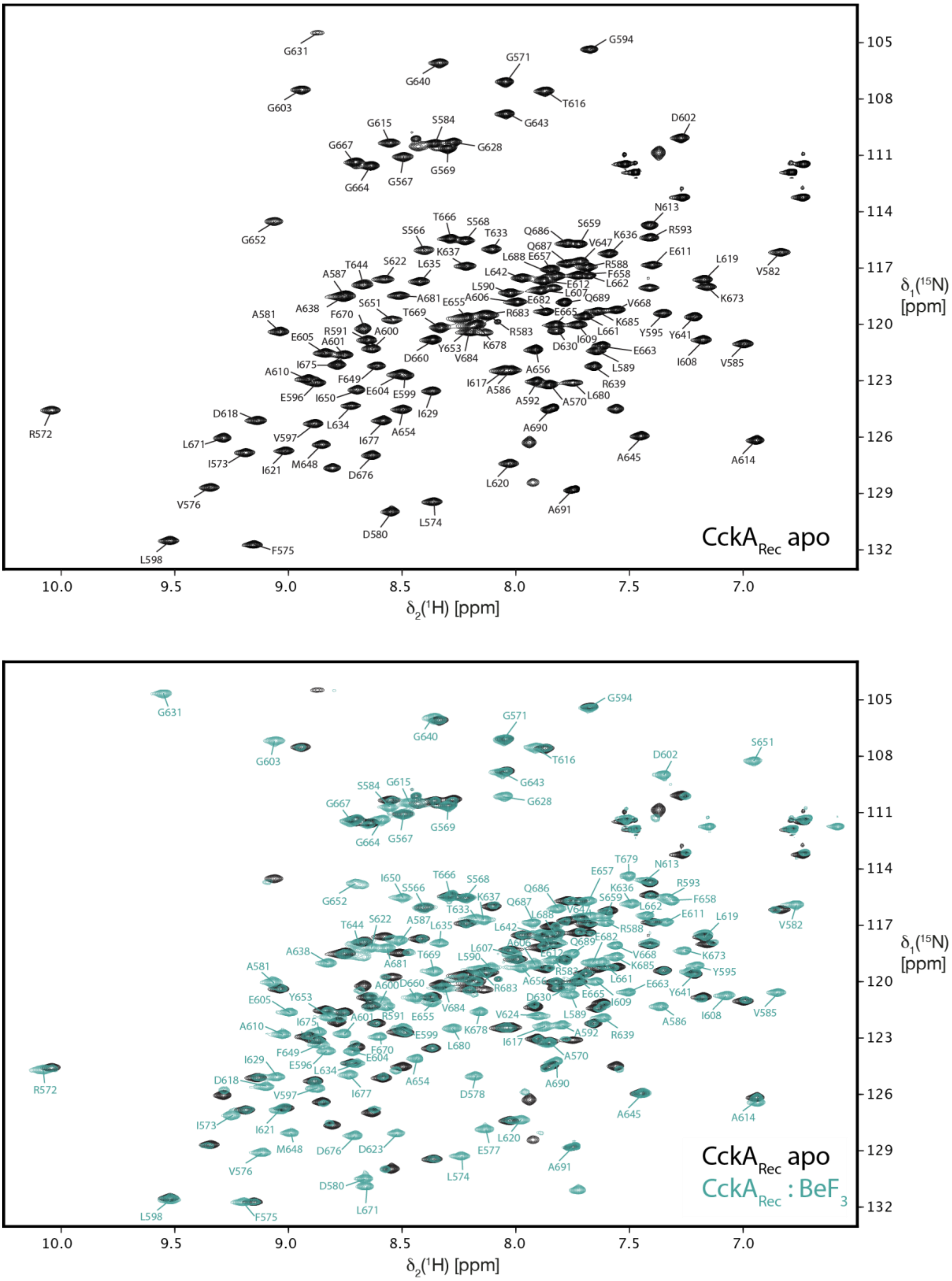

Supplementary Fig. 4. NMR resonance assignments of apo and BeF_3^--activated CckA^{Rec}. The top panel shows the 2D [^{15}N,^{1}H]-HSQC spectrum of 0.8 mM CckA^{Rec} recorded at 25°C in 25 mM MES pH 6.8 with 100 mM NaCl and 5 mM $MgCl_2$ in 95%/5% H_2O/D_2O. The sequence-specific resonance assignments are indicated. The bottom panel shows the same NMR spectrum of apo CckA^{Rec} as in the top panel (black resonances) overlaid with the 2D [^{15}N,^{1}H]-

HSQC spectrum of 0.4 mM CckA^{Rec} in the presence of 10 mM BeF_3^- (bluegreen resonances). Sequence-specific resonance assignments are indicated for the higher populated activated BeF_3^- bound state of CckA^{Rec}

a

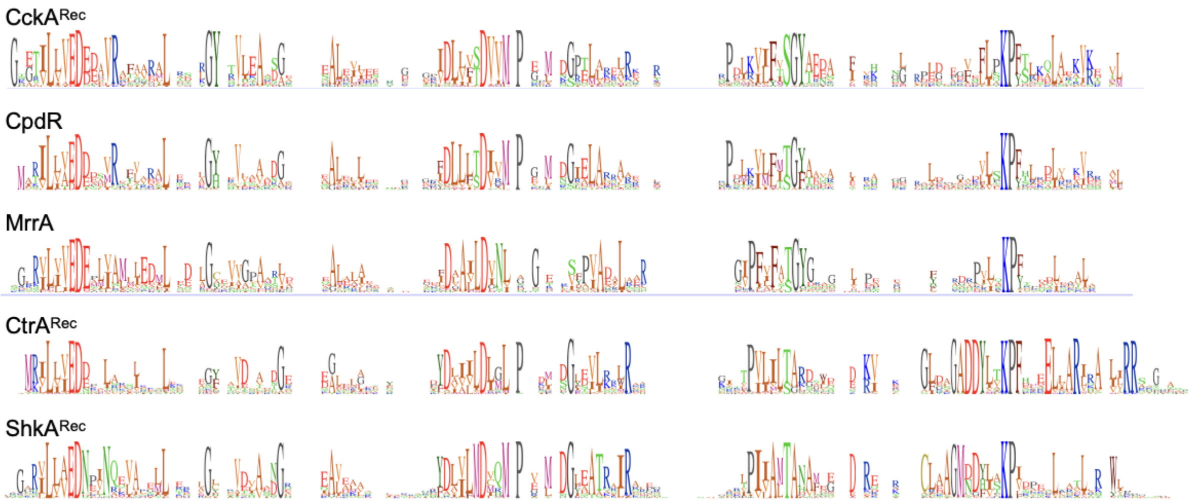

b

log(E) score of sequence/motif match

			Rec domain			HMM Rec motif				
organism	protein	UNIPROT	type	PDB	FATGUY motif	CckA^{Rec}	CpdR	MrrA	CtrA^{Rec}	ShkA^{Rec}
C. cresc	CckA	Q9X688	HHK	6tne	FISGYAE	-43	-33	-11	-16	-16
C. cresc	CpdR	Q9AA62	single		FITGFAA	-32	-49	-12	-20	-22
C. cresc	MrrA	Q9A428	single		FASGYGE	-14	-14	-49	-9	-10
C. cresc	CtrA	P0CAW8	RR			-19	-24	-13	-51	-24
C. cresc	ShkA	Q9ABT2	HHK *)	6qrj		-14	-20	-12	-27	-52
B. subtilis	Spo0F	P06628	single	2ftk	IMTAYGE	-26	-31	-17	-24	-24
R. meliloti	Sma011	Q930Y6	single	2lpm	FATGYGS	-15	-16	-47	-13	-11
S. melonis	SdrG	A0A0D1M	single	5ieb	FATGGSD	-10	-12	-44	-8	-6
P. aerug	PA1611	Q9I3B1	HHK	7c1j		-16	-22	-14	-29	-54
S. cerevisiae	SLN1	P39928	HHK	2r25		-14	-16	-6	-16	-37
A. thaliana	AHK5	Q3S4A7	HHK	4euk		-14	-18	-9	-17	-43
A. thaliana	CKI1	O22267	HHK	3mmn		-12	-12	-6	-13	-30
E. coli	RcsC	P0DMC5	HHK *)	2ayz		-22	-26	-11	-22	-35

Supplementary Fig. 5. Sequence logos for five distinct Rec$_{inter}$ groups and correlation (E-values) of selected sequences against derived HMM motifs.
(a) Rec logos as derived from the alignment of homologous sequences retrieved by HMMER. The names of the founder sequences are indicated. For details see Material and Methods.
(b) HMMSCAN E-scores of full-length sequences against the HMM Rec motifs corresponding to the logos shown in (A). Orange background: E < 1e-40, dark grey: E < 1e-30, light grey:E < 1e-25. *): C-terminal domain of the two Rec domains.

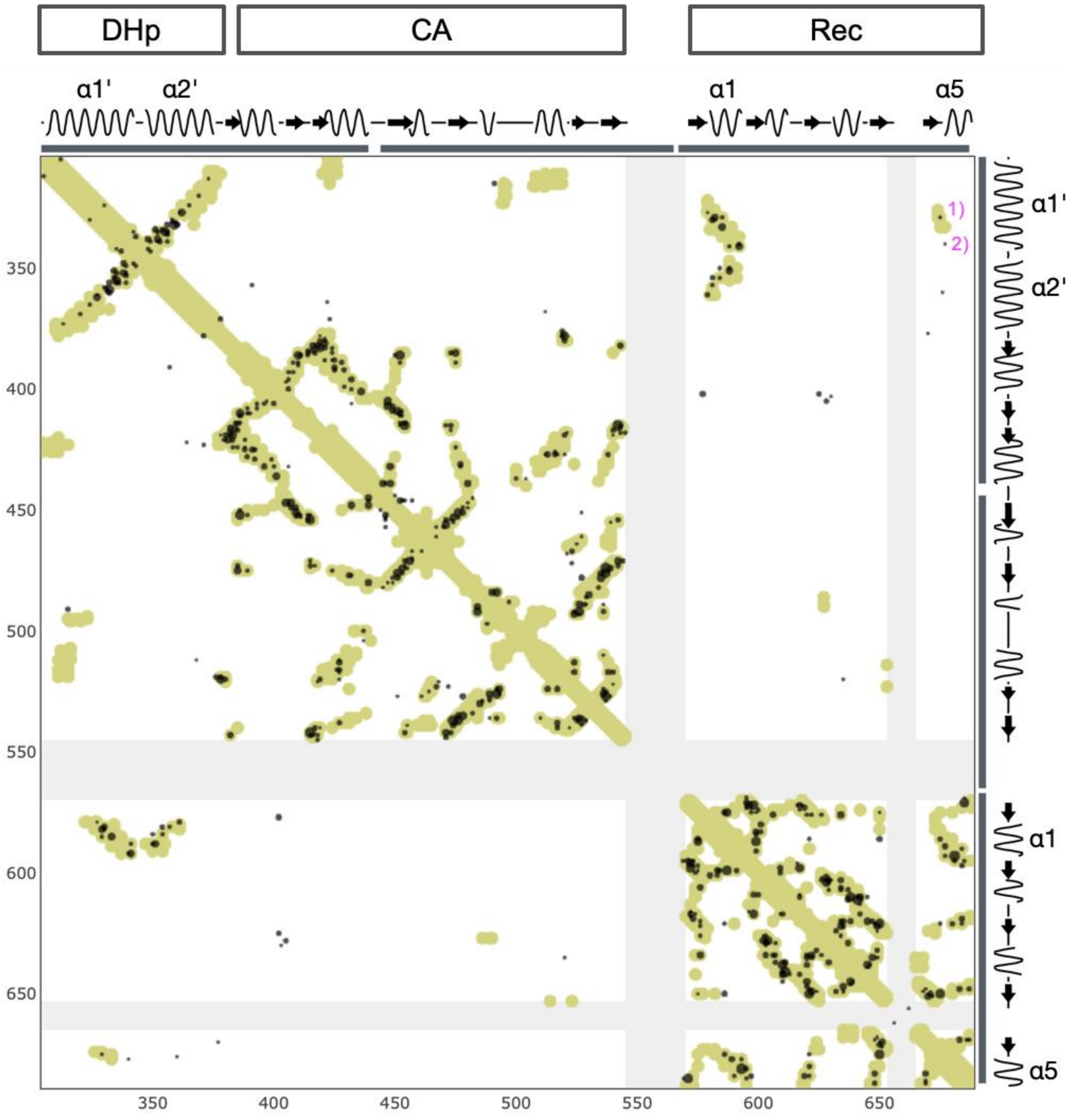

Supplementary Fig. 6. HHK co-variation analysis. EVcoupling matrix based on 8067 sequences retrieved with CckA query sequence and an E-value of 1e-60. All peaks (scaled according to score) with a probability >95% are shown. Yellow dots indicate contacts with a distance < 6Å in the CA_DHp_Rec phosphotransfer model of CckA (Fig. 5). Some helices of the DHp domain (α1', α2') and the Rec domain (α1 to α2) are labeled on the axes. Significant Rec / DHp (top right part of the matrix) covariation values are found almost exclusively between Rec-α1 and the α1', α2' DHp helices (see the zoom-in of Fig. 9). The peaks marked with 1) and 2) correspond to T329/I675 and H340/I677 (distance 10 Å), respectively, and involve Rec β5-α5. All other inter-domain correlations are probably spurious or indirect, since all their distances are > 20 Å

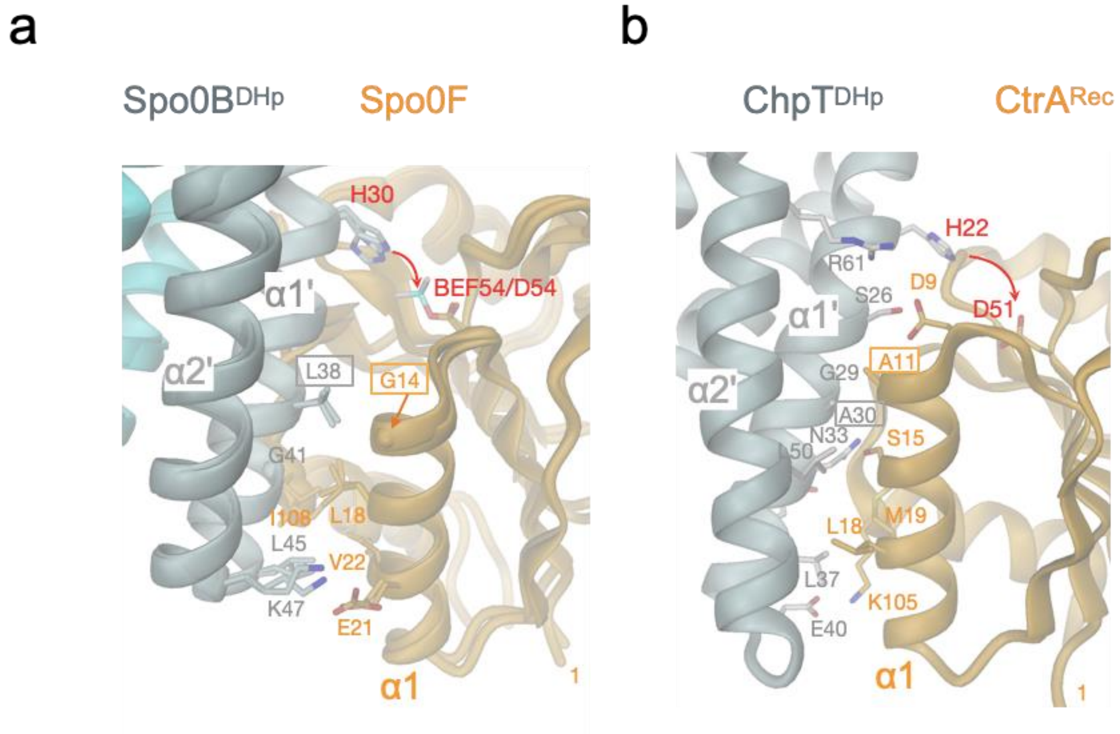

Supplementary Fig. 7. Structural comparison of DHp / Rec association in Spo0B/Spo0F and ChpT/CtrA. Cartoon representation (DHp, grey/aquamarine; Rec, orange) with interface residues shown in full. Active histidine and aspartate residues participating in the phosphotransfer are linked by a red arrow.

(a) Structure of Spo0B^{DHp}/Spo0F (1F51) superimposed onto the virtually identical Spo0B^{DHp}/BeF_3^--Spo0F (2FTK) complex. As a visual guide, the boxed residues are homologous to the boxed residues in panel b.

(b) Structure of ChpT^{DHp}/CtrA^{Rec} (4QPJ). This figure used for comparison is identical to Fig. 5b.

Supplementary Table

Supplementary Table 1. Amide proton linewidth of $CckA^{Rec}$ residues in the inactive and active state.

**Residue**	**Linewidth of amide proton (Hz)**	
	**native $CckA^{Rec}$**	**BeF_3^- modified $CckA^{Rec}$**
L574	22.3	20.6
V576	24.9	21.4
V582	24.8	18.4
V585	19.9	15.4
G603	24.0	23.4
I608	18.6	16.8

Supplementary Table 2. Primers used to obtain the recombinant plasmids used in this study .

**ID**	**Name**	**Sequence (5’>3’)**	**Length**	**Tm* (°C)**	**Exp Ta** (°C)**
**1**	Q5SDM_$CckA^{Rec}$_F	CACCATCACCATCACGGTTCTGGTTCTGG CGCCGGCCGCATCCT	44	75.3	72.0
**2**	Q5SDM_$CckA^{Rec}$_R	TCAATGGTGATGATGGTGGTGCTACG CCGCCTGCAGCTGCTGCTT	45	76.3	72.0
**3**	Q5SDM_$CckA^{Rec}$_N-histag_F	AAGGAGATATACCATATGGGCCATCAC CATCACCATCACGGT	42	68.0	72.0
**4**	Q5SDM_$CckA^{Rec}$_ N-histag_R	TCAATGGTGATGATGGTGGTGCTA CGCCGCCTGCAGCTGCTGCTT	45	76.3	72.0

* Tm = calculated melting temperature ** Exp Ta = annealing temperature used experimentally
